# Maize-legume intercropping achieves yield advantages by improving leaf functions and dry matter partition

**DOI:** 10.1186/s12870-023-04408-3

**Published:** 2023-09-19

**Authors:** Zhidan Fu, Ping Chen, Xiaona Zhang, Qing Du, Benchuan Zheng, Huan Yang, Kai Luo, Ping Lin, Yiling Li, Tian Pu, Yushan Wu, Xiaochun Wang, Feng Yang, Weiguo Liu, Chun Song, Wenyu Yang, Taiwen Yong

**Affiliations:** 1 College of Agronomy, Sichuan Engineering Research Center for Crop Strip Intercropping System/ Key Laboratory of Crop Ecophysiology and Farming System in Southwest, Ministry of Agriculture and Rural Affair, Sichuan Agricultural University, Huimin Road 211, Wenjiang District, Chengdu, 611130 China; 2 School of Life Science and Engineering, Southwest University of Science and Technology, Mianyang, 621010 Sichuan China; 3 Crop Research Institute, Sichuan Academy of Agricultural Science, Chengdu, 610066 People’s Republic of China; 4 Institute of Ecological and Environmental Science, College of Environmental Science, Sichuan Agricultural University, Chengdu, 611130 China

**Keywords:** Intercropping, Maize, Peanut, Soybean, Leaf functional trait, Yield advantage

## Abstract

Intercropping can obtain yield advantages, but the mechanism of yield advantages of maize-legume intercropping is still unclear. Then, we explored the effects of cropping systems and N input on yield advantages in a two-year experiment. Cropping systems included monoculture maize (*Zea mays* L.) (MM), monoculture soybean (*Glycine max* L. Merr.) (MS), monoculture peanut (*Arachis hypogaea* L.) (MP), maize-soybean substitutive relay intercropping (IMS), and maize-peanut substitutive strip intercropping (IMP). N input included without N (N0) and N addition (N1). Results showed that maize’s leaf area index was 31.0% and 34.6% higher in IMS and IMP than in MM. The specific leaf weight and chlorophyll a (chl a) of maize were notably higher by 8.0% and 18.8% in IMS, 3.1%, and 18.6% in IMP compared with MM. Finally, N addition resulted in a higher thousand kernels weight of maize in IMS and IMP than that in MM. More dry matter accumulated and partitioned to the grain, maize's averaged partial land equivalent ratio and the net effect were 0.76 and 2.75 t ha^−1^ in IMS, 0.78 and 2.83 t ha^−1^ in IMP. The leaf area index and specific leaf weight of intercropped soybean were 16.8% and 26% higher than MS. Although soybean suffers from shade during coexistence, recovered growth strengthens leaf functional traits and increases dry matter accumulation. The averaged partial land equivalent ratio and the net effect of intercropped soybean were 0.76 and 0.47 t ha^−1^. The leaf area index and specific leaf weight of peanuts in IMP were 69.1% and 14.4% lower than in the MP. The chlorophyll a and chlorophyll b of peanut in MP were 17.0% and 24.4% higher than in IMP. A less dry matter was partitioned to the grain for intercropped peanut. The averaged pLER and NE of intercropped peanuts were 0.26 and -0.55 t ha^−1^. In conclusion, the strengthened leaf functional traits promote dry matter accumulation, maize-soybean relay intercropping obtained a win–win yield advantage, and maize-peanut strip intercropping achieved a trade-off yield advantage.

**Supplementary Information:**

The online version contains supplementary material available at 10.1186/s12870-023-04408-3.

## Introduction

Agriculture production faces great challenges, such as increasing farmland productivity with limited resources to feed the continuously growing global population [1]. Intercropping, defined as two or more crops grown in the same land for the whole or a part of their growing period [2], has been used worldwide because of its high farmland productivity [3], efficient use of nutrients [4], better control of diseases and pests [2, 5], and its possibility for providing a sustainable agricultural development [6].

The yield advantages of intercropping systems are mainly based on the complementary or competitive use of resources [7]. However, the yield advantage of maize varies when intercropped with different legumes [3]. Although maize-soybean and maize-peanut intercropping achieves a land equivalent ratio (LER) of higher than one, maize yield increased while soybean and peanut yield decreased [7]. Reasonable N input in intercropping will improve the grain yield advantages [8]. For instance, the maize-legume intercropping benefits more from the complementary effect than the selection effect without N addition, but N fertilization led to a more robust yield advantage by shifting the complementary effect to the selection effect [7, 9, 10]. Although intercropping cereal with legumes can benefit cereal by strengthening the symbiosis nitrogen fixation of legumes and increasing soil N pool [11, 12], N input and cropping systems affected the N fixation ability and yield advantage of legumes in the intercropping [8, 13]. High N input inhibits the symbiosis of nitrogen fixation and decreases the grain yield of legumes, but the competitive use of N by cereal can alleviate the inhibiting effect [14]. Compared with wheat-faba bean intercropping, the grain yield and biological N fixation of faba bean improved in maize-faba bean intercropping [15]. To date, some studies have revealed the yield advantages mechanism of maize-legume intercropping from the standpoint of ecology [7, 16]. However, the yield advantages mechanism of maize-legume intercropping in physiology is still poorly understood, especially from leaf functions to yield formation and yield advantages in intercropping.

Biomass accumulation is the basis of yield, and the crop leaf functional traits affect biomass production. The leaf functional traits, e.g., leaf area (LA), leaf area index (LAI), specific leaf weight (SLW), chlorophyll content, and chloroplast ultrastructure, are used to assess the characteristics of crop growth on a temporal scale [17–19]. On the temporal and spatial scales, relative growth rates (RGR) help evaluate crop growth in different periods [20]. Besides, the crop growth environment plays a crucial role in photosynthesis, such as shading affecting the leaf morphology, structure, function, and biomass accumulation [21, 22]. Compared with normal light, shading increases soybean leaf chloroplast number, grana lamella thickness, and photosynthetic pigment per unit mass while decreasing the net photosynthetic rate and the chloroplast and starch grain size [18]. After maize harvest, the full sunshine condition is beneficial to increase the aboveground biomass, leaf thickness, and chlorophyll content of the intercropped soybean [23]. The recovery growth promotes biomass accumulation and improves soybean grain yield [19].

Previous studies have focused on ecology and agronomy aspects to investigate the yield advantage of intercropping [7, 16, 24], but few studies evaluate the leaf functional traits and dry matter partition on the yield advantage in maize-legume intercropping. Therefore, the objectives of this study were (1) to investigate the effects of cropping systems and N addition on crop leaf functional traits, e.g., leaf area index, specific leaf weight, and chloroplast ultrastructure, (2) to analyze the effect of cropping systems and N addition on crops dry matter accumulation and partition (3) to reveal relationships between the crop leaf functional traits, dry matter accumulation and partition, and yield advantage in maize-legume intercropping.

## Material and methods

### Site description, experimental design and sampling

The field experiment was conducted in Renshou County in 2017–2018 (29°60’ N, 104°00’ E), Sichuan Province, Southwest China. The climate of the field region was subtropical monsoon humid, with an average annual temperature of 17.4 °C, rainfall of 1009.4 mm, and sunshine of 1196.6 h. Before planting, soil fertility was investigated. The soil pH, organic matter, total N, total P, and total K were 8.18, 14.19 g kg^−1^, 1.22 g kg^−1^, 1.95 g kg^−1^, and 26.06 g kg^−1^. The maize (Xianyu 335), shade-tolerant soybean (Nandou 12), and shade-tolerant peanut (Tianfu 18) were used as experimental crops for the two-year located experiment.

A two-factor experiment was conducted with N input levels and cropping systems. The N input levels included N addition (N1) and without N (N0). In N addition treatments, urea was used as the N fertilizer, and the rate of fertilizer used in the current study was according to the local production requirement. The N fertilizer for monoculture cropping was 80 kg N ha^−1^ for legumes and 240 kg N ha^−1^ for maize. The N fertilizer for the intercropping was half of the monoculture cropping because a replacement intercropping with a replace ratio of 0.5 was used. Namely, the N fertilizer of intercropping system was 160 kg N ha^−1^, including 40 kg N ha^−1^ for legumes and 120 kg N ha^−1^ for maize (Table 1). The per-plant fertilizer was the same for the same crop in intercropping and monoculture.

**Table 1:** Nitrogen application levels

Cropping systems	Treatment	N rates (kg·N ha^−1^)	Base fertilizer (kg·N ha^−1^)	Topdressing (kg·N ha^−1^)	
				At V6	At V12
MM	N0	0	0	0	0
	N1	240	80	80	80
IMS/IMP	N0	0	0	0	0
	N1	160	80	40	40
MS/MP	N0	0	0	―	―
	N1	80	80	―	―

*MM* Monoculture maize, *IMS* Maize-soybean relay intercropping, *IMP* Maize-peanut strip intercropping, *MS* Monoculture soybean, *MP* Monoculture peanut, *V6* The six-leaf stage of maize, *V12* The twelve-leaf stage of maize, *N0* Without N, *N1* N addition

In the N addition treatment, the N fertilizer for intercropping and monoculture maize was divided into base fertilizer (80 kg N ha^−1^) and topdressing (the rest of the N fertilizer). The topdressing was separated into two equal parts and applied at the V6 and V12 stages of maize (Table 1). In intercropping, the legumes base fertilizer (40 kg N ha^−1^) was mixed and applied with maize topdressing at the V12 stage of maize. In the N addition treatment of monoculture legumes, the N fertilizer for monoculture soybean and peanut was used as the base fertilizer at 80 kg ha^−1^. The P (calcium superphosphate) and K (potassium chloride) fertilizers were used as base fertilizers at 120 kg P_2_O_5_ ha^−1^ and 100 kg K_2_O ha^−1^ for crops. The base fertilizer was strip placement 5 cm away from the seeds when sowing crops. The topdressing of maize was strip placement 5 cm away from the plants.

**Fig. 1:**
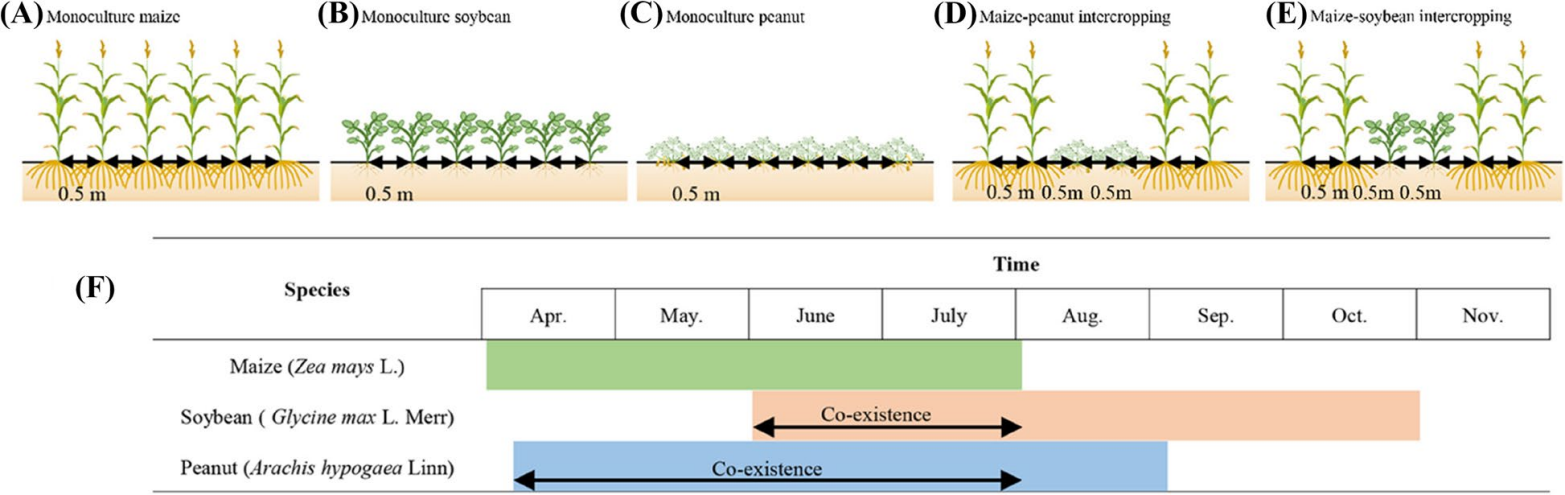
Schematic diagram of cropping system. Monoculture maize (MM), monoculture soybean (MS), monoculture peanut (MP), maize-soybean relay intercropping (IMS), maize-peanut strip intercropping (IMP)

Cropping systems included monoculture maize (MM), monoculture soybean (MS), monoculture peanut (MP), maize-soybean substitutive relay intercropping (IMS), and maize-peanut substitutive strip intercropping (IMP) (Fig. 1), with 2:2 row proportions under replacement series. The component species are almost simultaneous sowing in the strip intercropping systems, but the maturing species is interplanted with seeds of the following species in relay intercropping systems [25]. Three replicates for each treatment resulted in a total of thirty plots. The plot size was 6.0 m (width) × 5.8 m (length). An equal row spacing (0.5 m) planting method was adopted for all the cropping systems (Fig. 1). In all crop rows, one seedling per hole. The plant spacing was 0.2 m, and the density of maize was 100,000 plants ha^−1^ for M and 50,000 plants ha^−1^ for IMS and IMP. Maize was manually sown on April 8, 2017, and April 4, 2018, and harvested on August 4, 2017, and July 31, 2018. The plant spacing was 0.1 m, and the density of monoculture legumes (MS or MP) was 200,000 plants ha^−1^ and 100,000 plants ha^−1^ for intercropped legumes. Soybean was manually sown on June 10, 2017, and June 4, 2018, and harvested on November 1, 2017, and November 3, 2018. Peanut was manually sown on April 10, 2017, and April 9, 2018, and harvested on August 10, 2017, and August 31, 2018.

### Sampling and measurements

#### Leaf area index and specific leaf weight

Three individual plants from each plot were collected at the silking stage (R1) and milk stage (R3) of maize, the fifth trifoliolate stage (V5) and the full pod stage (R4) of soybean, and the beginning bloom stage (R1) and the full seed stage (R6) of peanut in the middle section of the middle row.

Maize leaf area was calculated as length multiplied by width and coefficient *K*, and the *K* is 0.75 [19]. Four leaf discs (diameter = 1.2 cm) were punched from each of the third leaves from the top of the soybean, and ten leaves were sampled. One leaf disc (diameter = 1.2 cm) was punched for each of the third leaves from the top of the top in peanut, and twenty leaves were sampled. The leaf discs and the sample leaf were over-dried at 75 °C to constant. Then the dry weight of the leaf was determined, and calculate leaf area. The leaf area was calculated as follows:

1 $$LA=\frac{L{A}_{\mathrm{disc}}}{{DM}_{\mathrm{disc}}} \times {DM}_{\mathrm{total}} \left({\mathrm{m}}^{2}\right)$$

where *LA*, *LA*_*disc*_, *DM*_*disc*_, and *DM*_*total*_ are the leaf area, leaf area of the discs, dry matter of the discs, and total leaf dry weight.

The observed and expected leaf area index (LAI) [26] was calculated as follows:

2 $$LA{I}_{\mathrm{obs}}=\frac{L{A}_{\mathrm{obs}\_\mathrm{A}}+L{A}_{\mathrm{obs}\_\mathrm{B}}}{Land\;area}$$

where *LAI*_*obs*_, *LA*_*obs_A*_, *LA*_*obs_B*_, and *Land area* are the observed leaf area index of the intercropping system, the observed leaf area of species A, the observed leaf area of species B, and the land area occupied by crops.

3 $$LA{I}_{\mathrm{exp}}=\frac{L{A}_{\mathrm{exp}\_\mathrm{A}}+L{A}_{\mathrm{exp}\_\mathrm{B}}}{Land\;area}$$

where *LAI*_*exp*_, *LA*_*exp_A*_, *LA*_*exp_B*_, and *Land area* are the expected leaf area index of the intercropping system, the expected leaf area of species A, the expected leaf area of species B, and the land area occupied by crops.

4 $$L{A}_{\mathrm{exp}\_\mathrm{A}}=L{A}_{\mathrm{mono}\_\mathrm{A}}\times {p}_{\mathrm{A}} {(\mathrm{m}}^{2})$$

5 $$L{A}_{\mathrm{exp}\_\mathrm{B}}=L{A}_{\mathrm{mono}\_\mathrm{B}}\times {p}_{\mathrm{B}} {(\mathrm{m}}^{2})$$

where *LA*_*mono_A*_, *LA*_*mono_B*_, *p*_*A*_, and *p*_*B*_ are the leaf area of monoculture species A, monoculture species B, the land sharing ratio of species A, and the land sharing ratio of species B.

The specific leaf weight (SLW) [27] was calculated as follows:

6 $$\text{SLW} = \frac{{DM}_{\mathrm{total}}}{\text{LA}} (\mathrm{mg c}{\mathrm{m}}^{-2})$$

where *DM*_*total*_ and *LA* are the total leaf dry weight and area.

#### Chlorophyll and chloroplast ultrastructure

Leaf samples of three individual plants from each plot were collected at the R1 and R3 stages of maize (ear leaf) [28], at the V5 and R4 stages of soybean (the third leaf from the top) [29, 30], and at the R1 and R6 stages of peanuts (the third leaf from the top) [31]. The leaf chlorophyll was extracted and determined using the method of Arnon [32]. Samples for chloroplast ultrastructure observation were fixed with a mixture of 3% glutaraldehyde and embedded to make semi-thin sections. The semi-thin sections were optically positioned, and ultrathin sections were stained with uranyl acetate and lead citrate, then examined with a transmission electron microscope (TEM; HITACHI, H-600IV, Japan) [18].

#### Measurements of dry matter and yield

Plant samples were collected at the V12, R1, R3, and R6 stages of maize, the V5, R2, R4, R6, and R8 stages of soybean, and the R1, R2, R4, and R6 stages of peanut. Three plants were sampled from each plot. These plant samples were separated into different organs: stem, leaf, and grain. Then, samples were oven-dried to constant weight at 75 °C and weighed.

Relative Growth Rate (RGR) is defined as growth in terms of a rate of increase in size per unit of size. The mean RGR over an interval of time between *t*_*1*_ and *t*_*2*_ is usually calculated as shown in the following formula [33]:

7 $$\mathrm{RGR}=\frac{{log}_{e}{W}_{2}-{log}_{e}{W}_{1}}{{t}_{2}-{t}_{1}}$$

where *W*_*2*_ and *W*_*1*_ are the dry matter at the sampling dates two and one, and *t*_*2*_ and* t*_*1*_ are the sampling dates, respectively.

At the mature stage, twenty consecutive plants of maize, soybean, and peanut were harvested in the middle row of each plot, and yield components were investigated. The net effect (NE) was used to assess the yield advantage of intercropping [7, 34], and the NE of two species of intercropping systems was calculated as follows [35]:

8 $$NE \left(\mathrm{tonne}\;per\;ha\right)=N{E}_{1}+N{E}_{2}$$

where *NE*, *NE*_1_, and *NE*_2_ are the net effect of intercropping, species one, and species two.

9 $$N{E}_{\mathrm{i}}={\mathrm{Y}}_{{\mathrm{obs}}_{\_}\mathrm{i}} -{\mathrm{Y}}_{{\mathrm{exp}}_{\_}\mathrm{i}}$$

where *NE*_*i*_ is the net effect of intercropping species i, *Y*_obs_i,_ and *Y*_exp_i_ are the observed and expected yields of species i (tonne per ha).

10 $${Y}_{\mathrm{exp}\_\mathrm{i}}={M}_{\mathrm{i}}\times {p}_{\mathrm{i}}$$

where *M*_i_ and *p*_i_ are the monoculture cropping yield (tonne per ha) and farmland sharing ratio of species i (%).

The land equivalent ratio (LER) was used to determine the land use advantage in intercropping and calculated as follows [36]:

11 $${\text{LER}}={\text{pLER}}_{\mathrm{m}}+{\text{pLER}}_{\mathrm{L}}=\frac{{Y}_{\mathrm{i}\_\mathrm{m}}}{{Y}_{\mathrm{s}\_\mathrm{m}}}+\frac{{Y}_{\mathrm{i}\_\mathrm{L}}}{{Y}_{\mathrm{s}\_\mathrm{L}}}$$

where *pLER*_m_ and *pLER*_L_ are the partial land equivalent ratio of maize and legumes. *Y*_i_m_ and *Y*_s_m_ are maize grain yields in intercropping and monoculture cropping. *Y*_i_L_ and *Y*_s_L_ are legumes grain yields in intercropping and monoculture cropping.

### Statistical and analysis

All statistical analyses were conducted with R v 4.4.2 [37]. We conducted a one-way ANOVA to test the effects of the cropping system, N levels, and growing seasons on crops’ LAI, SLW, RGR, biomass, and yield components, as well as on the pLER and NE of the component crops and the intercropping system. The one-way ANOVA (Tukey HSD, *p* < 0.05) was performed with *HSD.test()* functions in the R package *agricolae* v 1.3–6 [38]. Figures were drawn by GraphPad Prism 9 (GraphPad Software, San Diego, California, USA).

## Results

### Leaf Area Index (LAI) and Specific Leaf Weight (SLW)

**Fig. 2:**
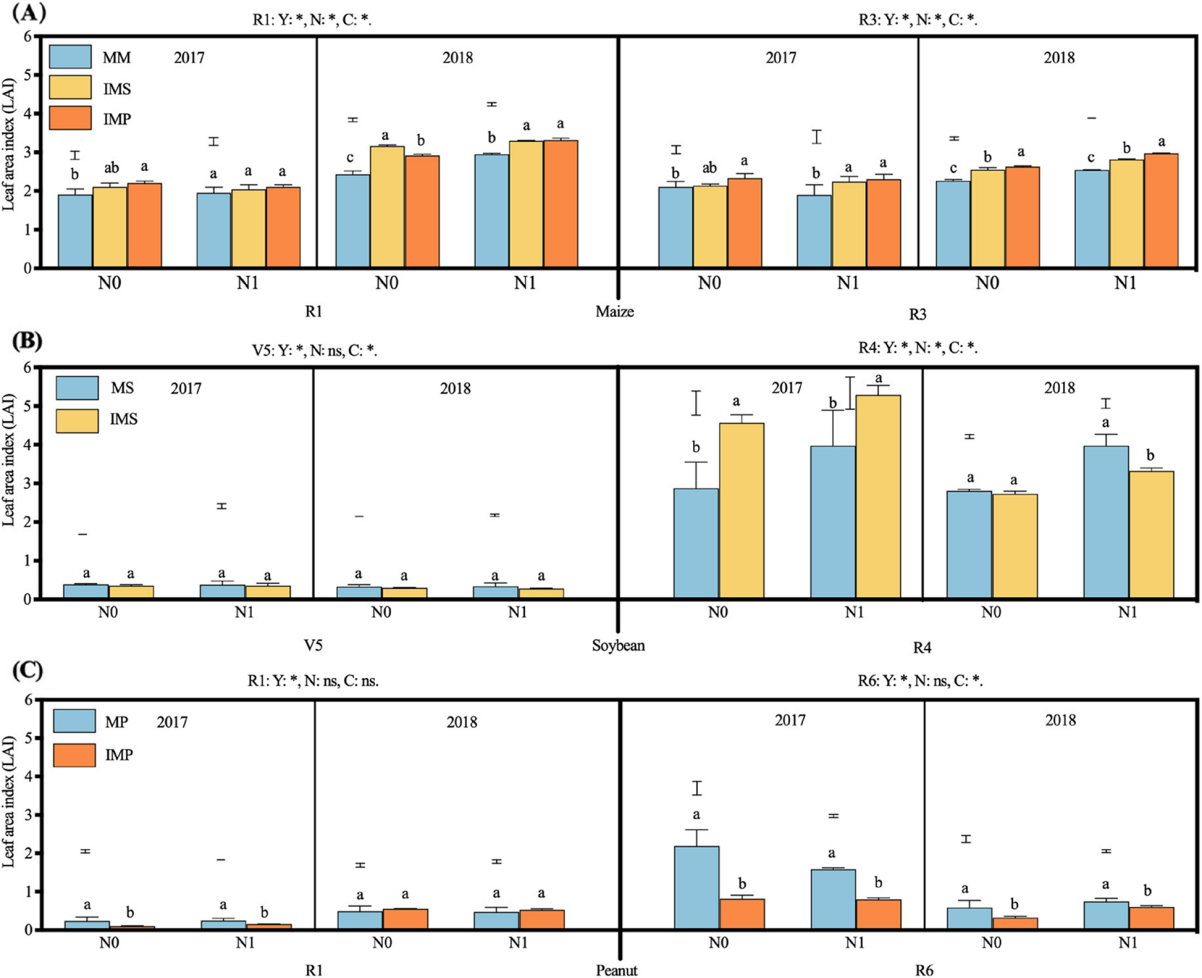
Effects of nitrogen input and cropping system on leaf area index (LAI). MM, monoculture maize, MS, monoculture soybean, MP, monoculture peanut, IMS, maize-soybean relay intercropping, IMP, maize-peanut strip intercropping. Panels **A** (maize), R1, the silking stage, and R3, the milk stage. Panels **B** (soybean), V5, the fifth trifoliolate stage, and R4, the full pod stage. Panels **C** (peanut), R1, the beginning bloom stage, and R6, the full seed stage. N0, 0 kg N ha^−1^, N1, 80 kg N ha.^−1^. Data were shown as mean with S.D. Different lower case letter donates significant difference between cropping systems under the same N input (Tukey HSD, *p* < 0.05). The Tukey HSD values were shown as standard bar above each N treatments. Results of the one-way ANOVA were displayed at the top of each panel, N, N input, C, cropping system, and ‘*’ and ‘ns’ represent significant and insignificant difference at the same growth stage (Tukey HSD, *p* < 0.05)

Nitrogen addition considerably enhanced maize’s LAI in 2018, but the LAI was independent of N addition in 2017 (Fig. 2A). The LAI of maize was 31.0% and 34.6% higher in IMS and IMP than in MM (2.26, mean of two years) (Fig. 2A). The LAI of soybean was independent of N addition at the V5 stage, but at the R4 stage, it was notably increased with N addition (Fig. 2B). The LAI of soybean in IMS (0.36, mean of two years) was 11.5% lower than in MS at the V5 stage. At the R4 stage, the leaf area index of intercropped soybean was 16.8% higher than that in MS in 2017, while that of intercropped soybean was lower than MS in 2018 (Fig. 2B). Similarly, the LAI of peanut was independent of N addition. Cropping seasons affected peanuts’ LAI. At the R1 stage, the LAI of peanuts was remarkably higher in 2018 than in 2017. In contrast, the LAI of peanut was greater in 2017 than in 2018 at the R4 stage (Fig. 2C). Moreover, the LAI of monoculture peanut (1.28, mean of two years) was 50.1% greater than that in IMP at the R6 stage.

N addition enhanced maize’s SLW in 2018, but it was insignificant in 2017 (Fig. S2A). The SLW of maize in IMS and IMP was 8% and 3.1% higher than that of MM (54.3 g m^−2^, mean of two years). Compared with MS, the SLW of intercropped soybean (48.3 and 50.7 g m^−2^, mean of two years) was notably higher by 7.9% under N0 and 7.1% under N1 at the R4 stage (Fig. S2B). Regarding peanut, intercropping significantly increased peanuts’ SLW in 2018. N addition remarkably enhanced SLW but intercropping resulted in a notable decrease in SLW at the R1 stage (Fig. S2C). The SLW of MP was 54.6% higher than that of IMP (21.84 g m^−2^, mean of two years) at the R6 stage.

### Chloroplast ultrastructure and chlorophyll (Chl) content

**Fig. 3:**
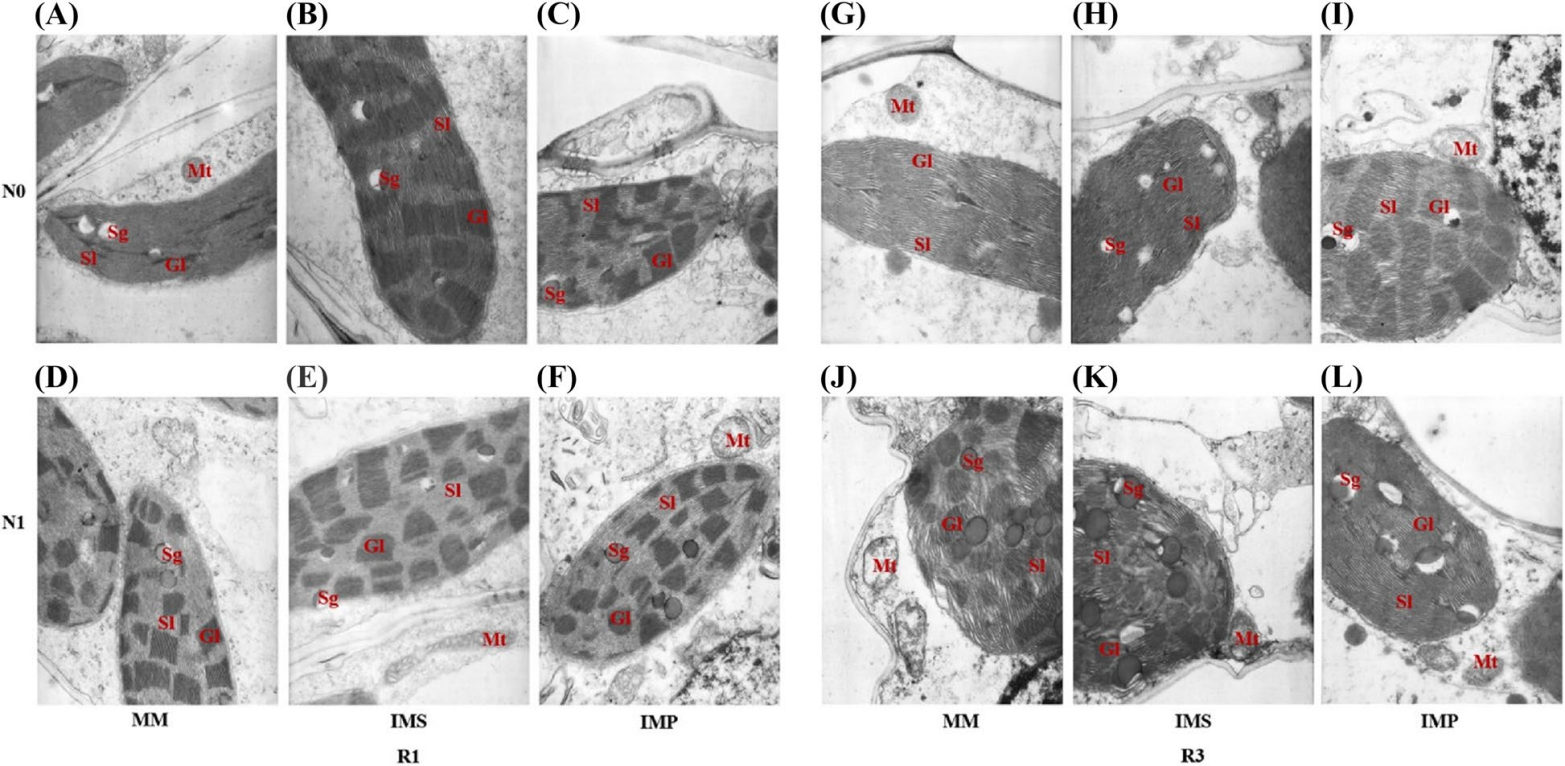
Effects of nitrogen input and cropping system on the chloroplast ultrastructure of maize functional leaf in 2018. R1, MM, monoculture maize, IMS, maize-soybean relay intercropping, and IMP, maize-peanut strip intercropping. N0, 0 kg N ha^−1^, and N1, 80 kg N ha^−1^. R1, the silking stage, and R3, the milk stage. Mt, Mitochondria, Gl, Grana lamella, Sl, Stroma lamella, Sg, Starch grain, Og, Osmiophilic granules. Panels **A**-**F**, Chloroplast ultrastructure of maize at the silking stage (R1), and panels **G**-**L**, Chloroplast ultrastructure of maize at the milk stage

**Fig. 4:**
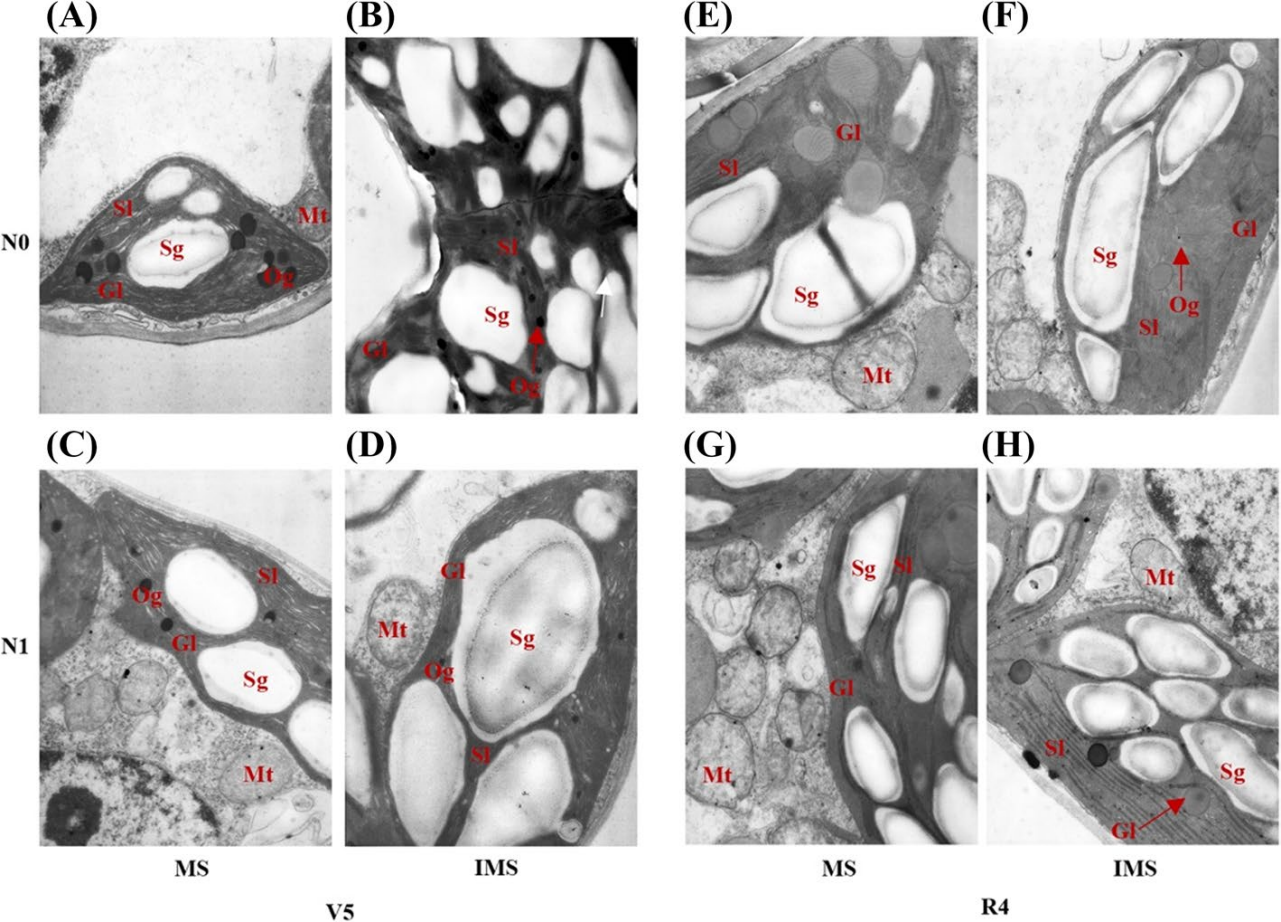
Effects of nitrogen input and cropping system on the chloroplast ultrastructure of soybean functional leaf in 2018. MS, monoculture soybean, and IMS, soybean in the maize-soybean relay intercropping. N0, 0 kg N ha^−1^, and N1, 80 kg N ha^−1^. V5, the fifth trifoliolate stage, and R4, the full pod stage. Mt, Mitochondria, Gl, Grana lamella, Sl, Stroma lamella, Sg, Starch grain, Og, Osmiophilic granules. Panels **A**-**D**, Chloroplast ultrastructure of soybean at the fifth trifoliolate stage, and panels **E**–**H**, Chloroplast ultrastructure of soybean the full pod stage

**Fig. 5:**
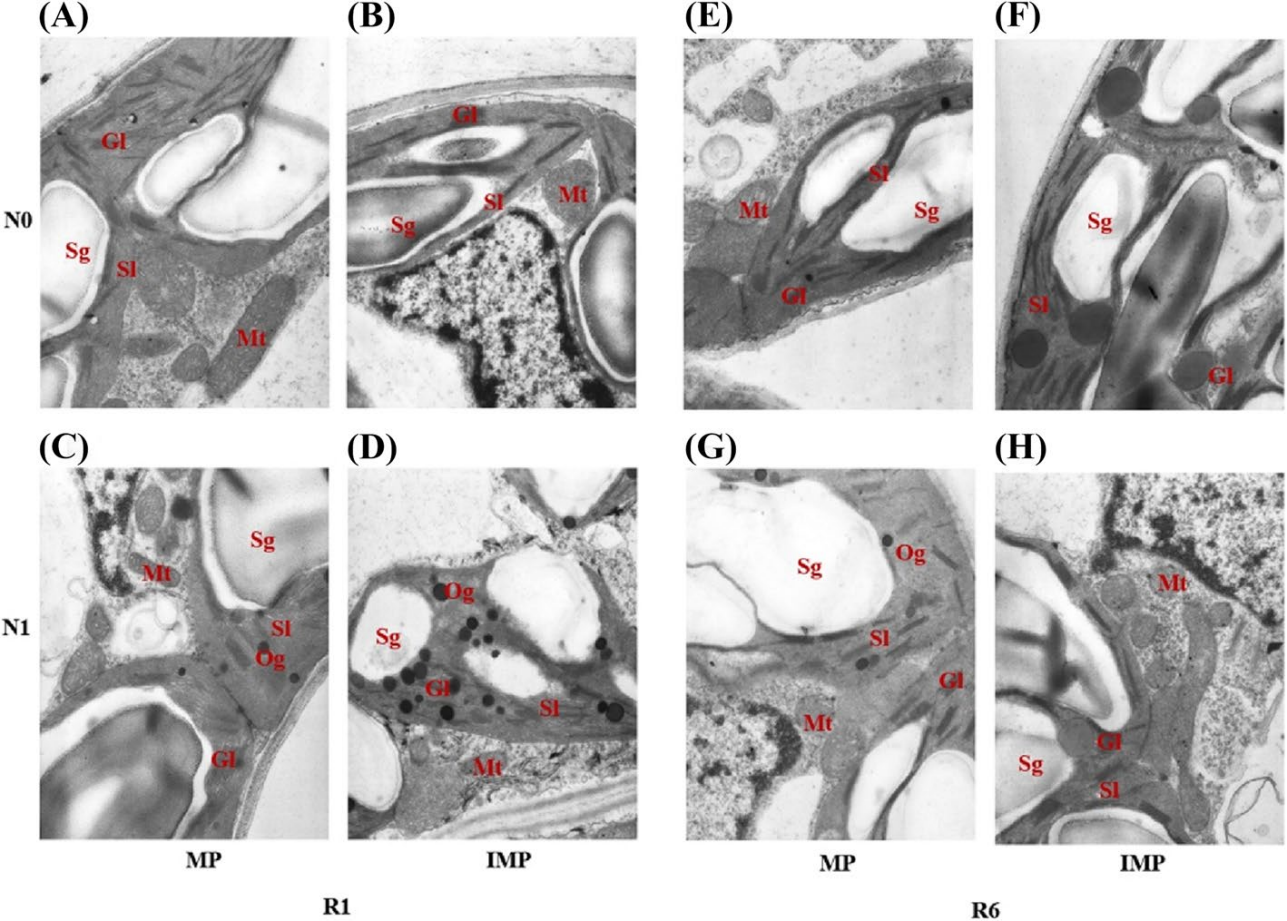
Effects of nitrogen input and cropping system on the chloroplast ultrastructure of peanut functional leaf in 2018. MP, monoculture peanut, and IMP, peanut in maize-peanut strip intercropping. N0, 0 kg N ha^−1^, and N1, 80 kg N ha^−1^. R1, the beginning bloom stage, and R6, the full seed stage. Mt, Mitochondria, Gl, Grana lamella, Sl, Stroma lamella, Sg, Starch grain, Og, Osmiophilic granules. Panels **A**-**D**, Chloroplast ultrastructure of peanut at the beginning bloom stage, and panels **E**–**H**, Chloroplast ultrastructure of soybean at the full seed stage

Nitrogen addition strengthened crop leaf physiological functional traits, such as promoting the development of chloroplast, increasing the number of chloroplast grana, and enhancing the accumulation of starch grana in the chloroplast (Figs. 3–5). The grana lamellae of maize leaves in IMS and IMP were thickened in MM (Fig. 3). In contrast to MM, the chloroplast volume of maize leaves was greater in IMS and IMP, and it was bigger in IMS than in IMP. In monoculture, the thylakoid of soybean leaves was elongated, and grana stacking was less at the V5 stage. In contrast, the thylakoid of soybean leaves in IMS was round and closely arranged with more starch grains, which occupied as much as 60% of the chloroplast (Fig. 4). Similarly, chloroplast volume and grana lamellae of soybean leaves were greater in IMS than in the monoculture at the R4 stage. The chloroplast volume of peanut leaves was lower in IMP than in the monoculture; however, more and larger grana lamellae and starch grains were observed in IMP than in the monoculture (Fig. 5). Similarly, the starch grains accounting for more than 60% of the chloroplast in peanut leaves (Fig. 5).

**Fig. 6:**
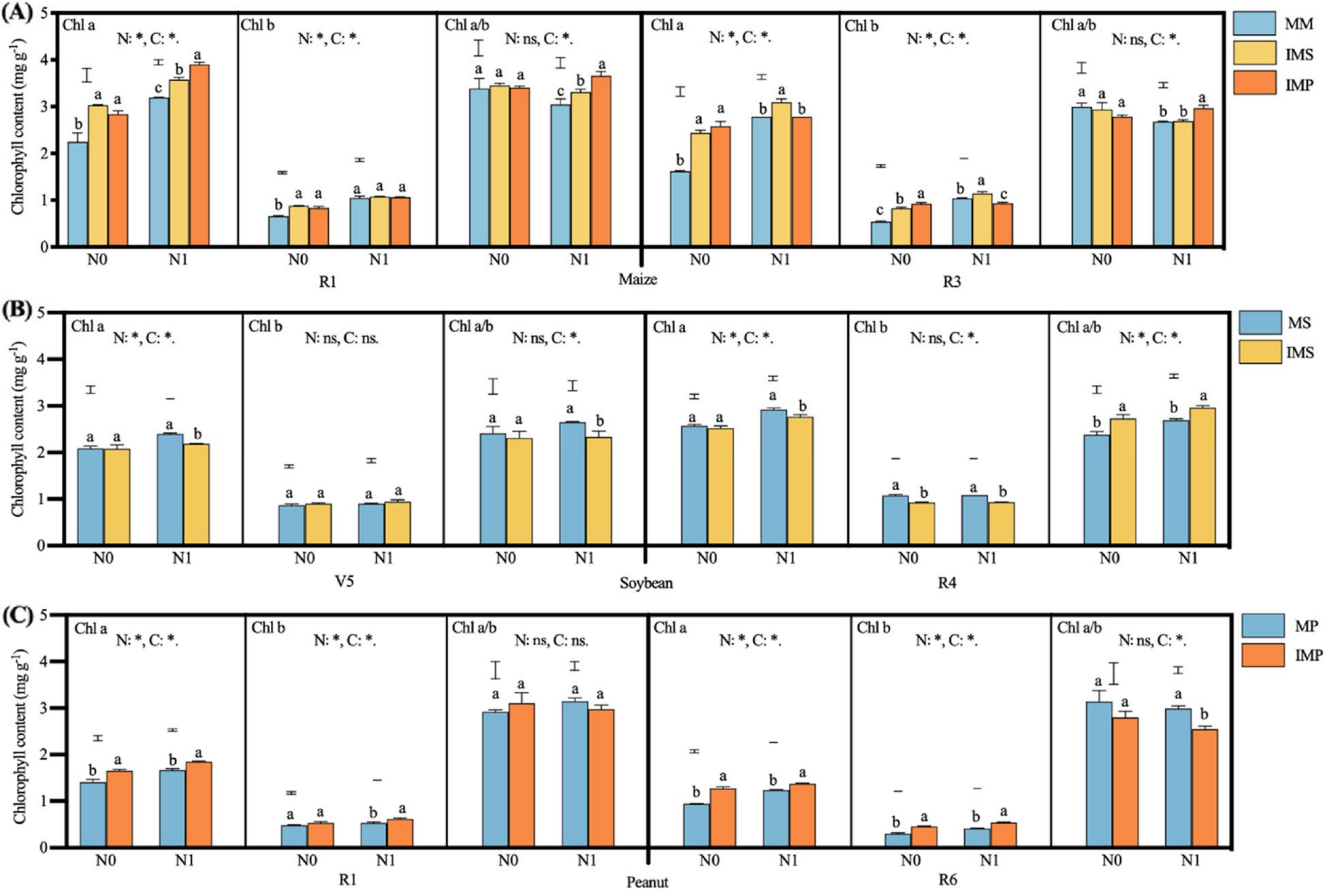
Effects of nitrogen input and cropping system on leaf chlorophyll content (2018). MM, monoculture maize, MS, monoculture soybean, MP, monoculture peanut, IMS, maize-soybean relay intercropping, IMP, maize-peanut strip intercropping. Chl a, Chlorophyll a, Chl b, Chlorophyll b, Chl a/b, Chlorophyll a/b ratio. Panels **A** (maize), R1, the silking stage, and R3, the milk stage. Panels **B** (soybean), V5, the fifth trifoliolate stage, and R4, the full pod stage. Panels **C** (peanut), R1, the beginning bloom stage, and R6, the full seed stage. N0, 0 kg N ha^−1^, N1, 80 kg N ha.^−1^. Data were shown as mean with S.D. Different lower case letter donates significant difference between cropping systems under the same N input (Tukey HSD, *p* < 0.05). The Tukey HSD values were shown as standard bar above each N treatments. Results of the one-way ANOVA were displayed at the top of each panel, N, N input, C, cropping system, and ‘*’ and ‘ns’ represent significant and insignificant difference at the same growth stage (Tukey HSD, *p* < 0.05)

Generally, nitrogen addition and intercropping notably affected crop leaf Chl a, Chl b, and Chl a/b (Fig. 6). The Chl a of maize in IMS and IMP was 18.8% and 18.6% higher than that in MM (2.72 mg g^−1^), while the Chl b was higher by 16.3% in IMS and 18.2% in IMP compared with MM (0.86 mg g^−1^), respectively (Fig. 6A). Although N addition decreased the Chl a/b ratio of maize, intercropping maize with legumes and N addition improved the Chl a/b ratio (Fig. 6A).

Regarding soybean (Fig. 6B), the Chl b content of soybean leaves in IMS at the R4 stage was 13.9% lower than that in the monoculture. The Chl a of soybean leaves in monoculture was 6.9% higher than in IMS with N addition (2.19 mg g^−1^). Independent of N addition, the Chl a/b ratio of soybean leaves was lower in IMS than in MS at the V5 stage, in contrast, the Chl a/b ratio was greater in intercropped soybean than in MS at the R4 stage (Fig. 6B). Focusing on peanuts (Fig. 6C), the contents of Chl a, Chl b, and Chl a/b decreased with the growth. The Chl a and Chl b of peanut leaves in IMP were 16.0% and 22.8% higher than the monoculture (1.45 and 0.47 mg g^−1^) with N addition. Besides, intercropping decreased the Chl a/b ratio of peanuts leaves at the R6 stage.

### Dry matter accumulation and partition

**Fig. 7:**
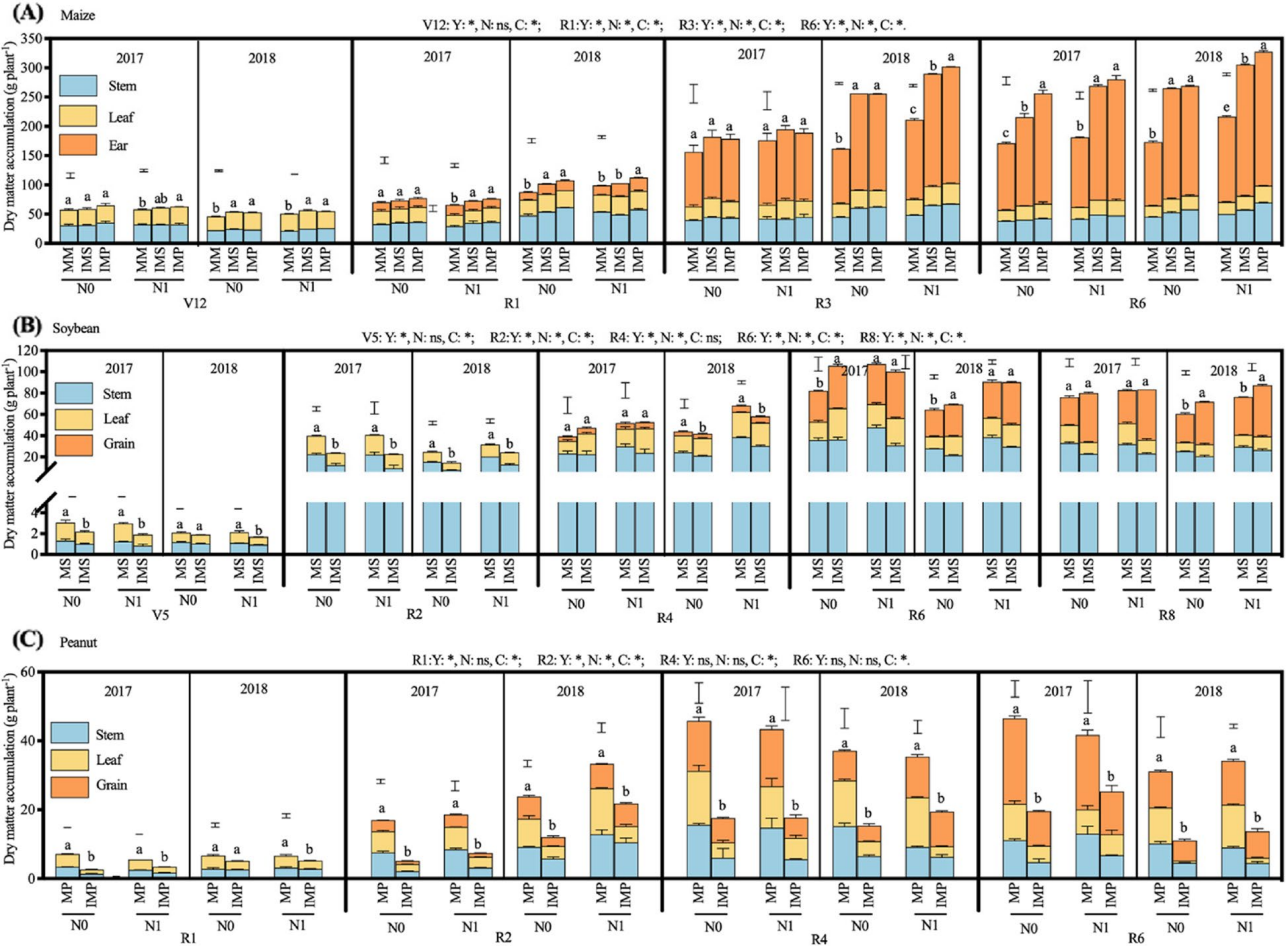
Effects of nitrogen input and cropping system on dry matter accumulation. MM, monoculture maize, MS, monoculture soybean, MP, monoculture peanut, IMS, maize-soybean relay intercropping, IMP, maize-peanut strip intercropping. Panels **A** (maize), V12, the twelfth leaf stage, R1, the silking stage, R3, the milk stage, R6, the maturity stage. Panels **B** (soybean), V5, the fifth trifoliolate stage, R2, the full bloom stage, R4, the full pod stage, R6, the full seed stage, and R8, maturity stage. Panels **C** (peanut), R1, the beginning bloom stage, R2, the beginning ped stage, R4, the full pod stage, R6, the full seed stage. N0, 0 kg N ha^−1^, N1, 80 kg N ha^−1^. Data were shown as mean with S.D. Different lower case letter donates significant difference between cropping systems under the same N input (Tukey HSD, *p* < 0.05). The Tukey HSD values were shown as standard bar above each N treatments. Results of the one-way ANOVA were displayed at the top of each panel, N, N input, C, cropping system, and ‘*’ and ‘ns’ represent significant and insignificant difference at the same growth stage (Tukey HSD, *p* < 0.05)

**Fig. 8:**
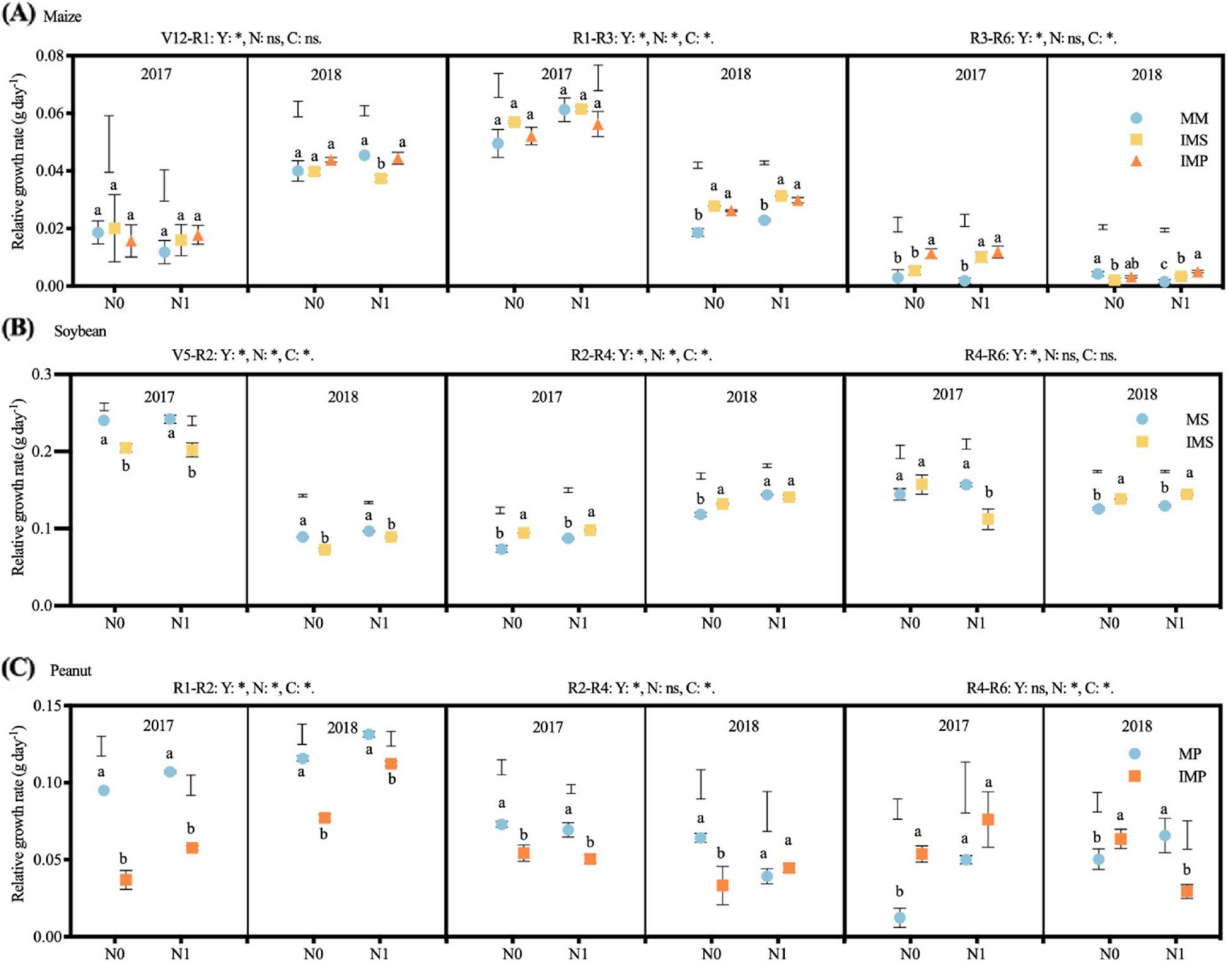
Effects of nitrogen input and cropping system on crops’ relative growth rate. MM, monoculture maize, MS, monoculture soybean, MP, monoculture peanut, IMS, maize-soybean relay intercropping, IMP, maize-peanut strip intercropping. Panels **A** (maize), V12-R1, from the twelfth leaf stage to silking stage, R1-R3, from the silking stage to milk stage, and R3-R6, from the milk stage to maturity stage. Panels **B** (soybean), V5-R2, from the fifth trifoliolate stage to full bloom stage, R2-R4, from the full bloom stage to full pod stage, and R4-R6, from the full pod stage to full seed stage. Panels **C** (peanut), R1-R2, the beginning bloom stage to beginning ped stage, R2-R4, from the beginning pod stage to full pod stage, R4-R6, from the full pod stage to full seed stage. N0, 0 kg N ha^−1^, N1, 80 kg N ha.^−1^. Data were shown as mean with S.D. Different lower case letter donates significant difference between cropping systems under the same N input (Tukey HSD, *p* < 0.05). The Tukey HSD values were shown as standard bar above each N treatments. Results of the one-way ANOVA were displayed at the top of each panel, N, N input, C, cropping system, and ‘*’ and ‘ns’ represent significant and insignificant difference at the same growth stage (Tukey HSD, *p* < 0.05)

The cropping season affected maize per-plant dry matter accumulation, except for the V12 stage, maize biomass was significantly higher in 2018 than in 2017. Except for the V12 stage, nitrogen addition notably affected maize per-plant dry matter accumulation (Fig. 7A). At the R6 stage, the per plant dry matter accumulation of maize in MM, IMS, and IMP increased by 15.3%, 20.0%, and 15.6% in N1 compared with N0, respectively. The dry matter accumulation of maize per plant in IMS and IMP 44.7% and 53.0% higher than that in MM (23.89 g plant^−1^, mean of two years), respectively. In zero N treatment, the biomass of MM was lower in 2018 than in 2017. With N addition, the biomass of intercropped maize was higher in 2018 than in 2017. (Figs. 7A and S3A, B).The accumulated dry matter of maize was mainly transported to the ear, and the dry matter of maize ear was greater in IMS and IMP than MM (Figs. 7A and S3A, B). With N addition, the RGR of maize was higher in both IMS and IMP than in MM at the R3-R6 period (Fig. 8A).

The biomass of soybean was significantly lower in 2018 than in 2017. Although soybean per plant dry matter was independent of N input at the V5 stage, N addition notably enhanced soybean dry matter accumulation at the reproductive growth stage (Fig. 7B). Compared with without N, soybean dry matter accumulation of MS (68.3 g plant^−1^, mean of two years) and IMS (79.6 g plant^−1^) was 16.5% and 12.5% higher in N1 at the R8 stage. The dry matter partitioned to the grain of soybean in IMS enhanced by N addition treatment, namely by 6.2% and 8.5% at the R4 and R6 stages, respectively (Fig. S3C, D). Meanwhile, the RGR of soybean was higher in 2018 than in 2017 at the V5-R2 and R2-R4 periods. The RGR of intercropped soybean was 4.9% and 5.5% higher in N1 than in N0 at the V5-R2 and R2-R4 periods (Fig. 8B). Finally, the average grain yield of intercropped soybean was 13.8% higher in N1 than in N0. The accumulation of soybean per plant dry matter was 35.5% lower in IMS than in MS (2.58 g plant^−1^, mean of two years) at the V5 stage (Fig. 7B). With soybean growth, a more robust increase of dry matter accumulation was obtained in IMS than in MS. The dry matter accumulation of soybean was 28.4% lower at the V5 stage and 14.4% higher in IMS at the R8 stage compared with MS (two-year average). Compared with MS, the dry matter allocation to soybean stem in IMS decreased, while dry matter allocation to soybean leaf and pod increased, especially in R6 and R8 (Fig. S3C, D). The RGR of soybean was 17.6% greater in MS than in IMS at the V5-R2 period; in contrast, it was 9.9% higher in IMS than in MS at the R2-R4 period (Fig. 8B).

The peanut per-plant dry matter accumulation was higher in 2018 than in 2017 at the R1 and R2 stages. The averaged per-plant dry matter of intercropped peanut (27 g plant^−1^) was 30% higher in N1 than in N0 at the R6 stage (Fig. 7C). More dry matter was partitioned to grain in intercropped peanut than in MP at the harvest stage (Fig. S3E, F). The RGR of intercropped peanut with N addition was 30% higher than without N (Fig. 8C). Intercropping harmed the dry matter accumulation of peanuts (Fig. 7C). Compared with MP (46.1 g plant^−1^), dry matter accumulation of peanuts was 48.1% lower in IMP in two years on average at the R6 stage. At the R1 stage, the dry matter allocation of peanuts to stems was fewer but more to leaves. As peanuts grew, dry matter partitioned to the pod gradually increased, but the partition to stem and leaf decreased (Fig. S3E, F). At maturity, dry matter partitioned to stem and pod gradually increased with the peanut growing in the monoculture, but less dry matter was partitioned to the pod in intercropped peanut. The RGR of peanuts (0.11 g day^−1^) was 58.1% significantly higher in MP than in IMP at the R1-R2 stage (Fig. 8C).

### Yield advantages of intercropping

Intercropping with legumes significantly increased maize kernel number and thousand kernels weight, but the magnitude of that increase depended on cropping seasons. In contrast, the kernel number of monoculture maize reduced with two years of zero N input (Table 2). In addition, maize kernel number and weight remarkably improved in 2017 and 2018 (Table 2). The per-plant kernel number of maize peaked in IMP (478.68 kernels), and the thousand kernels weight of maize in IMS (349.94 g) was the highest. Regarding soybean, the number of per-plant grains was notably 25% higher in IMS than in MS (98.89 seeds), and the number of per-plant grains was notably 13.5% higher in N addition compared with no N (104.25 g plant^−1^) (Table 2). The hundred-seed weight of soybean was improved when intercropped with maize or N addition. Generally, the number of per-plant peanuts grains was independent in N addition, but it was remarkably 49.9% lower in IMP than in MP (16.62 seeds). A lower hundred-seed weight of peanut was obtained when intercropped with maize, but it increased by 5.6% with N addition (60.66 g) (Table 2). In summary, intercropped maize with N addition obtained more kernels and greater seeds size; in contrast, the better growth of maize had disadvantageous effects on legumes growth and led to fewer seeds and smaller seeds size (Table 2).

**Table 2:** Effects of cropping seasons, N inputs and cropping system on crops yield components

Treatments			Maize		Soybean		Peanut	
Y	N	C	Kernels per ear	Thousand kernels weight (g)	Grain number per plant	Hundred seeds weight (g)	Grain number per plant	Hundred seeds weight (g)
2017	N0	Sole	325.2 c	345.1 c	96.3 b	17.0 a	18.6 a	61.9 a
	N0	IMS	403.8 b	361.7 a	124.4 a	17.4 a		
	N0	IMP	430.9 a	352.4 b			7.2 b	57.3 b
		*HSD-C*	9.01	6.79	5.13	1.77	1.44	2.52
	N1	Sole	333.8 b*	350.4 b*	114.3 a*	17.2 a*	19.9 a*N	64.7 a*N
	N1	IMS	456.6 a*	369.6 a*	130.2 a*	18.2 a*		
	N1	IMP	462.3 a*	357.6 bi			8.1 b*N	62.0 a*Y
		*HSD-C*	8.54	10.55	19.52	1.82	1.44	6.00
	*HSD-N*	Sole	1.94	4.5	17.50	1.88	1.41	6.24
	*HSD-N*	IMS	2.54	3.65	10.07	1.70		
	*HSD-N*	IMP	13.18	12.64			1.48	1.84
2018	N0	Sole	314.7 bY	303.3 cY	88.4 bY	18.0 aN	12.6 aY	61.8 aN
	N0	IMS	495.1 aY	326.1 bY	107.9 aY	17.9 aN		
	N0	IMP	492.9 aY	334.8 aY			9.1 bY	61.8 aY
		*HSD-C*	4.82	8.06	3.76	1.88	1.72	3.79
	N1	Sole	367.5 b*Y	307.0 biY	96.6 b*Y	18.8 a*N	15.5 a*Y	65.4 aiN
	N1	IMS	528.4 a*Y	342.3 a*Y	132.1 a*N	18.2 a*N		
	N1	IMP	528.6 a*Y	343.9 a*Y			8.9 biN	64.2 a*N
		*HSD-C*	7.24	6.57	3.05	1.41	1.76	2.03
	*HSD-N*	Sole	6.38	9.11	2.94	1.47	1.74	3.86
	*HSD-N*	IMS	6.25	6.46	3.84	1.82		
	*HSD-N*	IMP	3.62	2.86			1.74	3.86
*HSD-Y*	N0	Sole	3.60	7.40	5.26	1.01	1.52	4.25
*HSD-Y*	N0	IMS	3.47	5.72	3.58	2.37		
*HSD-Y*	N0	IMP	10.16	7.00			1.65	1.64
*HSD-Y*	N1	Sole	5.61	6.96	16.94	2.17	1.65	5.99
*HSD-Y*	N1	IMS	5.79	4.73	10.16	0.77		
*HSD-Y*	N1	IMP	9.43	10.90			1.57	2.08

*Y* Year, *N* N input, *C* cropping system. N0, without N input, N1, with N input. Sole, denoted sole maize, or sole soybean, or sole peanut. IMS, maize-soybean relay intercropping, IMP, maize-peanut strip intercropping. Data were shown as means. The one-way ANOVA Tukey HSD values were provided. *HSD-C*, the Tukey values of the cropping systems. Different lowercase letters (a-c) in the same column denoted significant differences among cropping systems with the same N input of each cropping season (Tukey HSD, *p* < 0.05). *HSD-N*, the Tukey values of N input. The asterisk (*) and *i* denoted significant and insignificant differences between N inputs within the same cropping system of each cropping season (Tukey HSD, *p* < 0.05). *HSD-Y*, the Tukey values of the cropping season. The uppercase letters Y and N denoted significant and insignificant differences between cropping seasons with the same N input and cropping system (Tukey HSD, *p* < 0.05)

Generally, Maize in IMP showed a higher pLER and net effect than IMS, but there were no differences between cropping systems. Compared with without N, N addition decreased pLER by 0.91% and enhanced net effect by 10.2% for maize in IMP (pLER_M_, 0.75, and net effect, 2.94 t ha^−1^ mean of two years) (Table 3). On average for two-year, soybean intercropped with maize showed a pLER and net effect of 0.75 and 0.44 t ha^−1^ in N0 and by 0.78 and 0.50 t ha^−1^ in N1 (Table 3). Although N addition led to a decrease in pLER by 0.1%, the net effect of soybean was 14.9% higher with N addition than without N (0.435 t ha^−1^) at two years average (Table 3). Although the pLER of peanut increased by N addition, the net effect of peanut decreased by N addition (Table 3). Finally, the LER (1.52) and net effect (3.21 t ha^−1^) of IMS were 25% and 40.6% higher than those of IMP (Table 3). In IMS, the LER and the net effect were 4.02% and 19.3% higher in N addition than without N (1.49 t ha^−1^).

**Table 3:** Yield advantages and LER of maize-legumes intercropping

	Treats		Maize				Soybean				Peanut				System	
Y	N	P	Y_exp_	Y_obs_	pLER	NE	Y_exp_	Y_obs_	pLER	NE	Y_exp_	Y_obs_	pLER	NE_P_	LER	NE
2017	N0	Sole	5.61				1.63				1.14					
	N0	IMS		7.30b	0.65b	1.69b		2.17	0.66	0.53					1.31a	2.22a
	N0	IMP		7.59a	0.68a	1.98a						0.41	0.36	-0.74	1.04b	1.24b
		*HSD-C*		0.12	0.01	0.13									0.13	0.35
	N1	Sole	5.85*				1.95*				1.29*					
	N1	IMS		8.44a*	0.72a*	2.59a*		2.37i	0.61i	0.41i					1.33ai	3.00a*
	N1	IMP		8.27a*	0.71ai	2.42ai						0.50*	0.39i	-0.78i	1.10bi	1.63bi
		*HSD-C*		0.37	0.03	0.39									0.11	0.55
	*HSD-N*	Sole	0.09				0.20	0.33			0.14	0.08				
	*HSD-N*	IMS		0.07	0.01	0.06			0.12	0.40					0.12	0.35
	*HSD-N*	IMP		0.38	0.04	0.41							0.10	0.19	0.12	0.55
2018	N0	Sole	4.77Y				1.59N				0.78Y					
	N0	IMS		8.07aY	0.85aY	3.30bY		1.93N	0.61N	0.34N					1.45bY	3.64bY
	N0	IMP		8.25 aY	0.86 aY	3.48 aY						0.56Y	0.36N	-0.22Y	1.23aY	3.26aY
		*HSD-C*		0.19	0.02	0.17									0.09	0.32
	N1	Sole	5.64*Y				1.81 *N				1.01*Y					
	N1	IMS		9.04a*Y	0.80a*Y	3.40aiY		2.40*N	0.66iN	0.59iN					1.47aiY	3.99aiY
	N1	IMP		9.09a*Y	0.81a*Y	3.45aiY						0.57iN	0.28*Y	-0.44*Y	1.09*N	3.01*Y
		*HSD-C*		0.19	0.03	0.29									0.11	0.47
2018	*HSD-N*	Sole	0.11				0.15				0.14					
	*HSD-N*	IMS		0.26	0.03	0.31		0.24	0.10	0.30		0.12			0.12	0.51
	*HSD-N*	IMP		0.08	0.02	0.14							0.06	0.16	0.07	0.25
*HSD-Y*	N0	Sole	0.09				0.17				0.15					
*HSD-Y*	N0	IMS		0.19	0.02	0.17		0.31	0.12	0.37					0.13	0.39
*HSD-Y*	N0	IMP		0.12	0.02	0.14						0.10	0.08	0.16	0.09	0.27
*HSD-Y*	N1	Sole	0.10				0.18				0.11					
*HSD-Y*	N1	IMS		0.19	0.03	0.26		0.27	0.10	0.34					0.11	0.48
*HSD-Y*	N1	IMP		0.37	0.04	0.41						0.09	0.08	0.19	0.11	0.55

*Y* Year, *N* N input, *C* Cropping system. N0, without N input, N1, with N input. Sole, denoted sole maize, or sole soybean, or sole peanut. IMS, maize-soybean relay intercropping, IMP, maize-peanut strip intercropping. Data were shown as means. The one-way ANOVA Tukey HSD values were provided. *HSD-C*, the Tukey values of the cropping systems. Different lowercase letters (a-c) in the same column denoted significant differences among cropping systems with the same N input of each cropping season (Tukey HSD, *p* < 0.05). *HSD-N*, the Tukey values of N input. The asterisk (*) and *i* denoted significant and insignificant differences between N inputs within the same cropping system of each cropping season (Tukey HSD, *p* < 0.05). *HSD-Y*, the Tukey values of the cropping season. The uppercase letters Y and N denoted significant and insignificant differences between cropping seasons with the same N input and cropping system (Tukey HSD, *p* < 0.05)

## Discussion

### Performances of maize in maize-soybean and maize-peanut substitutive intercropping

Due to the taller plant height of maize, maize dominates and shades legumes in the maize-legume intercropping systems, intercropping with legumes and N addition was beneficial to increasing the LAI and SLW of maize (Figs. 2 and S2). Soybean was sown at the V12 stage of maize, and maize was harvested at the R1 stage of soybean. Leaf trait plays a critical role in light use and biomass accumulation, such as LAI and SLW directly affect light intercept and photosynthesis [39]. A higher LAI denotes more leaf area on the unit farmland and greater light interception by crop. Meanwhile, a greater SLW indicates leaves are thicker. Amanullah [40] pointed out that high-yield cultivars obtain thicker leaves and higher photosynthetic capacities. Indeed, consistent results were observed that thicker leaves were associated with a more robust synthesis of starch in leaves. The maize leaf chloroplast volume improved and grana lamellae stacking thickened when intercropped with legumes and N addition (Fig. 3).

A lower N condition reduces leaf chlorophyll content, and thinner leaves usually obtain lower chlorophyll content [40]. In the current study, the chlorophyll of maize was higher in IMS and IMP than in monoculture maize (Fig. 6). But the leaf functional traits of maize in IMP were more robust than in IMS (Figs. 3 and 6). On the one hand, light is absorbed by chlorophyll and transformed into organic compounds in the chloroplast [41]. The photoreaction is localized in the internal chloroplast membrane, called thylakoid; thus, the structure and quantity of thylakoids are decisive in effective photosynthesis [42–44]. As light irradiance reduced, the numbers of chloroplasts and grana lamellae increased, while the shape of chloroplast changed from ellipse or olive to swollen oblate or spheroidal. On the other hand, the Chl a content has also been suggested as one of the most decisive factors; namely, increasing Chl a can increase the photosynthetic rate [45, 46]. When intercropped with legumes, maize enhanced the light capture and was used through increasing LAI and SLW, enhancing leaf chlorophyll content, and strengthening leaf photosynthetic product accumulation. Therefore, the strengthened light-use ability is the base of biomass accumulation and yield.

**Fig. 9:**
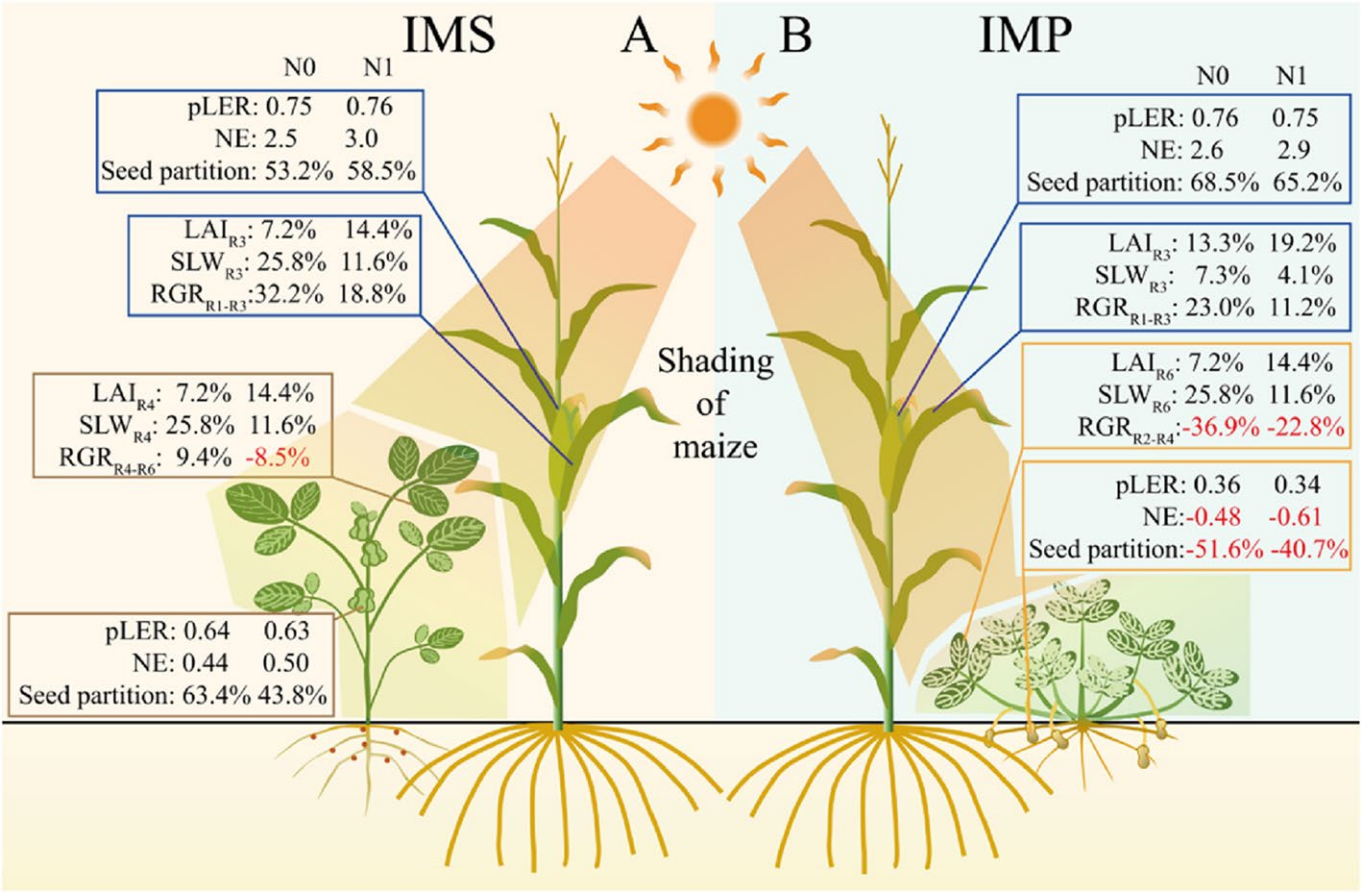
Model diagram of N inputs and cropping system on crops’ leaf functional traits and dry matter partition. Data of the partial land equivalent ratio (pLER) and net effect (NE) were shown as values (mean of two year) of the component crops under different N inputs. Data of the seed partition at the maturity stage, leaf area index (LAI), specific leaf weight (SLW), and relative growth rates (RGR) were shown as the relative changes (mean of two year) in intercropping compared with the corresponding monoculture cropping. The subscript denotes the corresponding growth stages of the crop

Indeed, the improved leaf traits enhanced maize’s relative growth ratio in intercropping systems (Fig. 9). Because of that, intercropped maize obtained a greater dry matter accumulation and grain yield than monoculture maize. Probably due to legume height being lower than maize, maize intercepted more light in intercropping than monoculture [47]. Although the belowground interactions increase maize biomass and grain yield in maize-peanut substitutive strip intercropping [48], the belowground interactions are independent of maize yield in the additive relay strip intercropping [49]. The different maize responses to the below ground are probably due to the wide interspecific distance in the maize-soybean intercropping (60 cm) than the maize-peanut intercropping (35 cm) [48, 49]. Wangiyana et al. [50] also found that leaves of red rice at anthesis much greener (indicating higher N) in intercropping with peanut resulting in much higher grain yield than in monocropped rice. Moreover, Nitrogen addition further enhances intercropping maize’s dry matter accumulation and grain yield. Intercropping sweet corn with peanut, presumably increasing N supply to sweet corn, was also reported to increase number of green leaves, biomass and cob weight of sweet corn per plant [51].

The kernel per ear and thousand kernels weight of maize increased when intercropped with legumes (Table 2). Although the kernel per ear of maize was higher in IMP than in IMS, the thousand kernels weight of maize was greater in IMS than in IMP. Sufficient rainfall facilitated acquisition of soil nutrients, while excessive rainfall at the grain filling stage (July 2018) also means a limitation on maize grains filling (Fig. S1). Therefore the kernel number of maize was greater in 2018 than in 2017, but the kernels weight of maize was lower in 2018 than in 2017 (Table 2). Besides, N addition notably increase the yield components of intercropped maize. Long-term intercropping enhances soil fertility and crop grain yield [4]. The improved nutrient condition is the belowground interaction mechanism of high-yielding of intercropping. Legumes can fix nitrogen and increase soil N pool, and peanut facilitates maize Fe and Zn nutrition in intercropping systems [52]. Therefore, interspecific facilitation improves crop nutritional status and promotes crop growth [52]. The consistent results were observed that the net effect and pLER of maize without N were 2.5 t ha^−1^ and 0.75 when intercropped with soybean, and N addition resulted in a greater net effect and pLER by 3.0 t ha^−1^ and 0.76 (Fig. 9). Although N addition led to a slight decrease in pLER of maize when intercropped with peanut, the NE of maize shift from 2.6 to 2.9 t ha^−1^ (Table 3).

### Physiological adjustment of the intercropped legumes under different shade times

The shorter component species suffer from the shading of the taller species in intercropping systems [19, 53]. Although the competitive use of light adversely affected the LAI and SLW of legumes during the coexistence period, the LAI and SLW of soybean can be recovered after the maize harvest (Figs. 2 and S2). In contrast, the penalty on peanuts’ LAI and SLW were not alleviated after the maize harvest (Figs. 2 and S2). The duration and intensity of shade in soybean and peanut differed, leading to a small peanut leaf area and a weak leaf functional trait. Therefore, soybean recovered after the coexistence and somewhat improved the leaf functional traits. The variation of legumes’ LAI and SLW was mainly caused by the shift of light environment in the intercropping systems. Shading decreases the LAI and SLW of intercropped soybean by reducing leaf size and thickness in the maize-soybean intercropping [18, 54]. After the maize harvest, the compensatory growth of soybean can eliminate the yield disadvantage [19]. Interestingly, although the recovered growth of soybean increase LAI and SLW, it was slightly different during the two cropping seasons. This was probably due to the less precipitation in 2017 than in 2018 (Fig. S1), which limited maize growth (Fig. 2A). Water deficiency will limit maize LAI and decrease grain yield [13]; in contrast, an alleviated competitive use of resources between maize and soybean results in a yield advantage of soybean [55].

The total chlorophyll of soybean significantly decreased when intercropped with maize, while the total chlorophyll of soybean was notably enhanced by N addition. The inference of cropping systems and N inputs on the chlorophyll mainly resulted from Chl a rather than Chl b (Fig. 6B). A higher chlorophyll contributes to utilizing light more efficiently, especially in a shaded environment [18]. Plants growing in the shade can optimize their light absorption efficiency by increasing pigment density per unit leaf area [56]. The increase in Chl b is most likely due to changes in the light-harvesting organization [57].

In contrast, intercropping and N addition notably enhanced peanuts’ Chl a and Chl b (Fig. 6C). In other words, peanuts suffer from a heavier shading than soybean when intercropped with maize, attributed to a relatively extended coexistence period (Fig. 1F). This adjustment reduced the respiratory demand to help compensate for the significantly decreased photosynthetic capacity of leaves [58]. Moreover, the Chl a/b of intercropped maize decreased through the quick increase in Chl a; N addition can alleviate the penalty on Chl a/b (Fig. 6A). The Chl a/b of the legume was reduced during the coexistence period through a quick increase in Chl b. The previous study indicates that the enhancement of Chl b is beneficial to increase the light harvest pigment complex, then improve the adaptability of plants to the shade condition [59].

Transmission electron microscopy showed that N addition could increase the chloroplast volume and thicken the grana lamellae of soybean and peanut (Figs. 4–5). At the V5 stage, the thylakoids of monoculture soybean were long, and grana lamellae decreased; in contrast, the thylakoids of intercropped soybean were round and starch grains closely arranged (Fig. 4). At the R4 stage, the chloroplast volume and grana lamellae of intercropped soybean increased while the arrangement was clear. Although the chloroplast volume of intercropped peanut decreased compared with monoculture peanut, the grana lamellae and starch grains of intercropped peanut increased (Fig. 5). The shading of maize led to more grana containing and thylakoids in peanut leaf. It is a critical shade-tolerant mechanism of plants by modulating the development of chloroplast and formating more numbers of thylakoids and grana as well as the grana lamellae [60]. At the later growth stage, chloroplast senescence happened, and the chloroplast shape changed from elliptical to spherical. Then, the accumulation of starch and the gradual disturbance of thylakoid, including distortion of granular arrangement, is accompanied by an increased number and size of translucent plastoglobuli [61]. The coexistence period of soybean was shorter than peanuts in intercropping systems (Fig. 1F). Especially peanuts suffer from the heavy shade of maize at the later growth stages (reproductive stages) rather than at the early stages (vegetative stages). Then, intercropped soybean obtained a more extended recovery growth than intercropped peanuts after the maize harvest.

The dry matter accumulation of intercropped soybean decreased at the V5 stage but increased at the R4 stage; in contrast, the dry matter accumulation of intercropped peanuts decreased. The variation of dry matter accumulation was consistent with the corresponding leaf traits (Figs. 2 and 7). The yield advantage of intercropping is affected by the component crop growth and dry matter partition [62, 63]. The previous study documented that shifts of leaf traits, e.g., LAI, SLW, and chlorophyll, increase photosynthesis and change the accumulation and partition of dry matter [64]. The RGR of intercropped soybean varied from -8.5% to 9.4% during the R4-R6 period; in contrast, the RGR of intercropped peanuts ranged from -22.8% to -36.9% (Fig. 9). The competitive use of resources affects the component crops’ growth and dry matter partition [22]. A less competitive component species usually captures few available resources, produces less dry matter due to weakening growth rates, and allocates less dry matter to the grain than the monoculture [3], ultimately affecting the yield advantages of intercropping. More dry matter was partitioned to seed for soybean; in contrast, the less dry matter was allocated to seed for peanut (Figs. 7 and S3). Firstly, the different responses of soybean and peanut leaf to shading removal lead to differences in dry matter accumulation. Secondly, the coexistence period of soybean was shorter than peanuts, leading to a more extended recovery growth. Thus, intercropped soybean could allocate more photosynthetic products to grain than monoculture soybean. However, more extended shade periods for peanuts, especially the heavy shading during the late reproductive stages, resulted in less dry matter partitioned to the seed. Therefore, intercropping shapes the characteristics of dry accumulation and partition of legumes (Fig. 9).

The per-plant grain number of intercropped soybean increased (Table 2), while the per-plant grain number and hundred seeds weight of intercropped peanut decreased (Table 2). Compared with 2017, sufficient rainfall benefited maize growth in 2018 (Fig. 7), which may result in heavy shading on legumes. A more extended coexistence period in peanuts than in soybean (Fig. 1) may limit peanuts’ light interception in intercropping (Figs. 2 and 5). Then, heavy shade with excessive rainfall from July to August 2018 led to fewer seeds and smaller seeds size of intercropped peanuts (Table 2). Although growth suppression happened due to stronger competition from the more dominant crop, its yield loss is also often reduced due to compensatory effects resulting from changes in morphology and functional traits [7, 65, 66]. Compared with monoculture, more erect leaves, greater specific leaf weight, and prolonged growth duration were reported in intercropping systems [7, 65, 66]. N addition increased soybean grain number, peanut grain number and hundred seeds weight (Table 2). Notably, a more extended recovery growth of soybean than peanut contributed to yield recovery in intercropping (Table 3). Although legume can meet about 50–60% of the N requirement for growth through biological nitrogen fixation, reasonable N input helps increases grain yield [67]. Finally, intercropped soybean obtained a net effect ranging from 0.44 to 0.50 t ha^−1^, but a negative value of net effect ranging from -0.48 to 0.61 t ha^−1^ was obtained for intercropped peanuts (Fig. 7).

## Conclusions

In the current study, intercropped maize obtained a yield advantage by strengthening leaf functional traits and dry matter partition. Namely, intercropped maize increases the leaf chloroplasts, grana, and grana lamellae, increasing chlorophyll and SLW to promote dry matter accumulation. Although the shade of maize in the coexistence period has adverse effects on soybean growth, the leaf functional traits, e.g., LAI, SLW, chlorophyll, and chloroplast, are strengthened in the recovered growth stages. Then, the intercropped soybean obtained a yield advantage. In contrast, the leaf functional trait indicates an irreversible penalty of maize’s heavy shade during the coexistence period on peanut growth can not be compensated during the recovered growth period. Finally, the yield disadvantage happened to intercropped peanuts. The land equivalent ratio and the net effect of maize-soybean intercropping ranges from 1.38 to 1.60 and 2.22 to 3.99 t ha^−1^, and from 0.89 to 1.13 and 1.24 to 3.26 t ha^−1^ for maize-peanut intercropping. Overall, maize-soybean relay intercropping obtains a win–win yield advantage, and maize-peanut strip intercropping achieves a trade-off yield advantage.

### Supplementary Information

**Additional file 1: Figure S1. **Weather condition of the experimental site.** Figure S2.** Effects of nitrogen input and cropping system on specific leaf weight (SLW). MM, monoculture maize, MS, monoculture soybean, MP, monoculture peanut, IMS, maize-soybean relay intercropping, IMP, maize-peanut strip intercropping. Panels A-B (maize), R1, the silking stage, and R3, the milk stage. Panels C-D (soybean), V5, the fifth trifoliolate stage, and R4, the full pod stage. Panels E-F (peanut), R1, the beginning bloom stage, and R6, the full seed stage. N0, 0 kg N ha-1, N1, 80 kg N ha-1. Data were shown as mean with S.D. Different lower case letter donates significant difference between cropping systems under the same N input (Tukey HSD, *p* < 0.05). The Tukey HSD values were shown as standard bar above each N treatments. Results of the one-way ANOVA were displayed at the top of each panel, N, N input, C, cropping system, and ‘*’ and ‘ns’ represent significant and insignificant difference at the same growth stage (Tukey HSD, *p* < 0.05).** Figure S3.** Effects of nitrogen input and cropping system on aboveground dry matter allocation. MM, monoculture maize, MS, monoculture soybean, MP, monoculture peanut, IMS, maize-soybean relay intercropping, IMP, maize-peanut strip intercropping. Panels A-B (maize), V12, the twelfth leaf stage, R1, the silking stage, R3, the milk stage, R6, the maturity stage. Panels C-D (soybean), V5, the fifth trifoliolate stage, R2, the full bloom stage, R4, the full pod stage, R6, the full seed stage, and R8, the maturity stage. Panels E-F (peanut), R1, the beginning bloom stage, R2, the beginning ped stage, R4, the full pod stage, R6, the full seed stage. N0, 0 kg N ha^-1^, N1, 80 kg N ha^-1^. Data were shown as mean with S.D.

## Data Availability

The original contributions presented in the study are included in this article. Further inquiries can be directed to the corresponding author.
