# Supplementary material for: Maize-legume intercropping achieves yield advantages by improving leaf functions and dry matter partition
Source: BMC Plant Biol. 2023 Sep 19;23:438. doi: 10.1186/s12870-023-04408-3 (PMC10507892; doi:10.1186/s12870-023-04408-3)
Supplement: Supplementary file 1 — Additional file 1: Figure S1. Weather condition of the experimental site. Figure S2. Effects of nitrogen input and cropping system on specific leaf weight (SLW). MM, monoculture maize, MS, monoculture soybean, MP, monoculture peanut, IMS, maize-soybean relay intercropping, IMP, maize-peanut strip intercropping. Panels A-B (maize), R1, the silking stage, and R3, the milk stage. Panels C-D (soybean), V5, the fifth trifoliolate stage, and R4, the full pod stage. Panels E-F (peanut), R1, the beginning bloom stage, and R6, the full seed stage. N0, 0 kg N ha-1, N1, 80 kg N ha-1. Data were shown as mean with S.D. Different lower case letter donates significant difference between cropping systems under the same N input (Tukey HSD, p < 0.05). The Tukey HSD values were shown as standard bar above each N treatments. Results of the one-way ANOVA were displayed at the top of each panel, N, N input, C, cropping system, and ‘*’ and ‘ns’ represent significant and insignificant difference at the same growth stage (Tukey HSD, p < 0.05). Figure S3. Effects of nitrogen input and cropping system on aboveground dry matter allocation. MM, monoculture maize, MS, monoculture soybean, MP, monoculture peanut, IMS, maize-soybean relay intercropping, IMP, maize-peanut strip intercropping. Panels A-B (maize), V12, the twelfth leaf stage, R1, the silking stage, R3, the milk stage, R6, the maturity stage. Panels C-D (soybean), V5, the fifth trifoliolate stage, R2, the full bloom stage, R4, the full pod stage, R6, the full seed stage, and R8, the maturity stage. Panels E-F (peanut), R1, the beginning bloom stage, R2, the beginning ped stage, R4, the full pod stage, R6, the full seed stage. N0, 0 kg N ha-1, N1, 80 kg N ha-1. Data were shown as mean with S.D. [file 12870_2023_4408_MOESM1_ESM.docx]

# Additional files 1

**Additional Figures l****egends**

**Figure. S1** Weather condition of the experimental site.

**Figure. S2** Effects of nitrogen input and cropping system on specific leaf weight (SLW).

**Figure. S3** Effects of nitrogen input and cropping system on aboveground dry matter allocation.


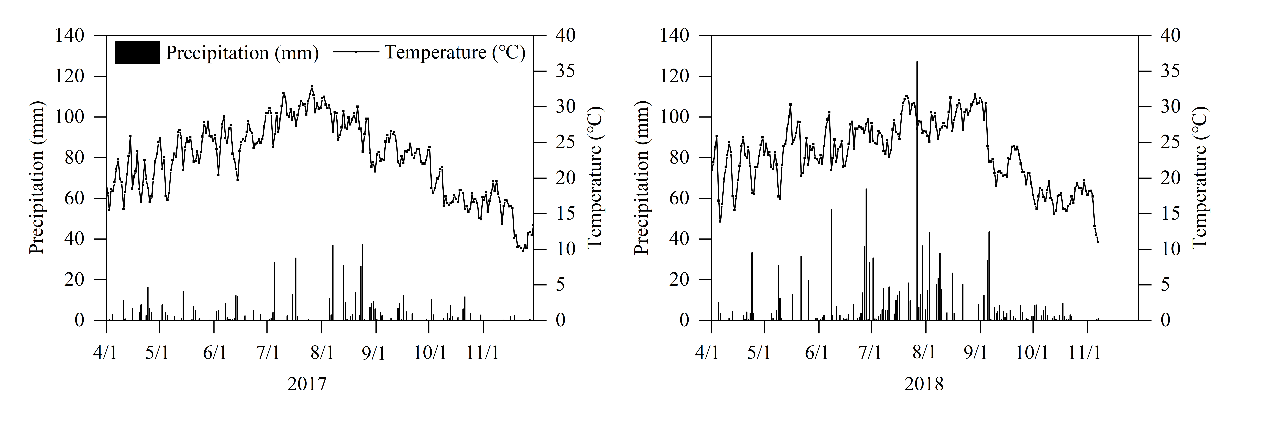


**Figure. S1** Weather condition of the experimental site.


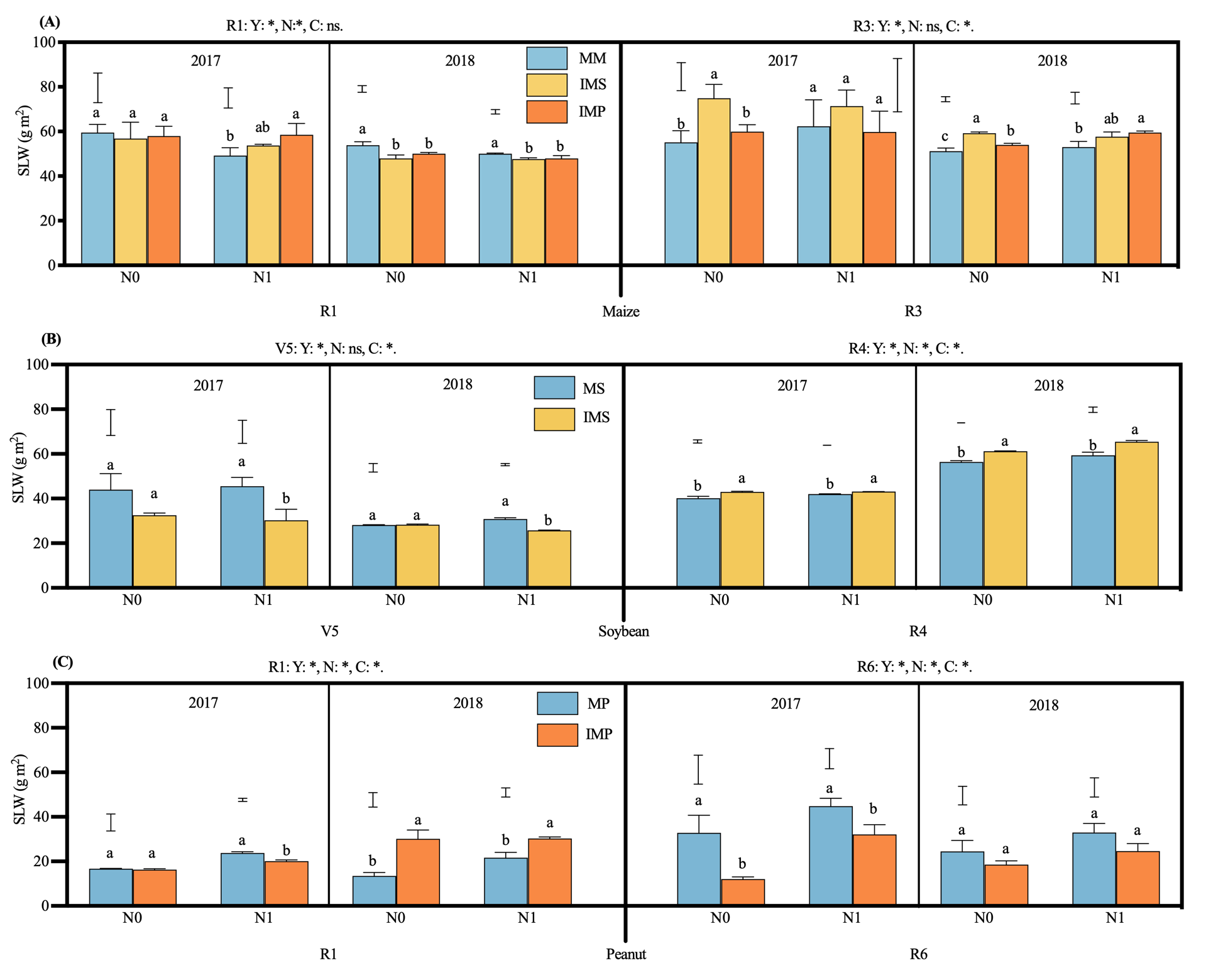


**Figure. S2** Effects of nitrogen input and cropping system on specific leaf weight (SLW). MM, monoculture maize, MS, monoculture soybean, MP, monoculture peanut, IMS, maize-soybean relay intercropping, IMP, maize-peanut strip intercropping. Panels A-B (maize), R1, the silking stage, and R3, the milk stage. Panels C-D (soybean), V5, the fifth trifoliolate stage, and R4, the full pod stage. Panels E-F (peanut), R1, the beginning bloom stage, and R6, the full seed stage. N0, 0 kg N ha-1, N1, 80 kg N ha-1. Data were shown as mean with S.D. Different lower case letter donates significant difference between cropping systems under the same N input (Tukey HSD, *p* < 0.05). The Tukey HSD values were shown as standard bar above each N treatments. Results of the one-way ANOVA were displayed at the top of each panel, N, N input, C, cropping system, and ‘*’ and ‘ns’ represent significant and insignificant difference at the same growth stage (Tukey HSD, *p* < 0.05).

**
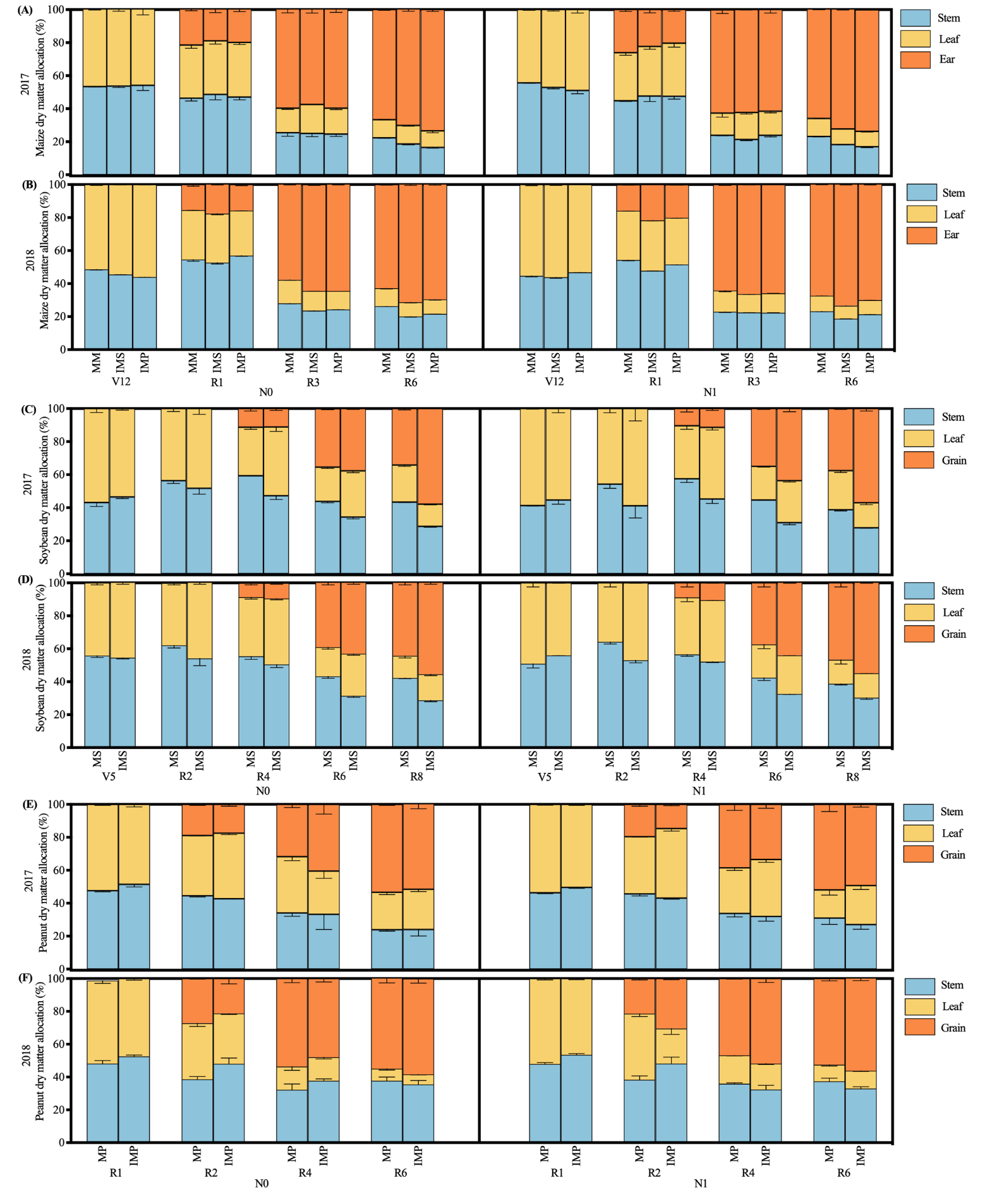
Figure. S3** Effects of nitrogen input and cropping system on aboveground dry matter allocation. MM, monoculture maize, MS, monoculture soybean, MP, monoculture peanut, IMS, maize-soybean relay intercropping, IMP, maize-peanut strip intercropping. Panels A-B (maize), V12, the twelfth leaf stage, R1, the silking stage, R3, the milk stage, R6, the maturity stage. Panels C-D (soybean), V5, the fifth trifoliolate stage, R2, the full bloom stage, R4, the full pod stage, R6, the full seed stage, and R8, the maturity stage. Panels E-F (peanut), R1, the beginning bloom stage, R2, the beginning ped stage, R4, the full pod stage, R6, the full seed stage. N0, 0 kg N ha^-1^, N1, 80 kg N ha^-1^. Data were shown as mean with S.D.
